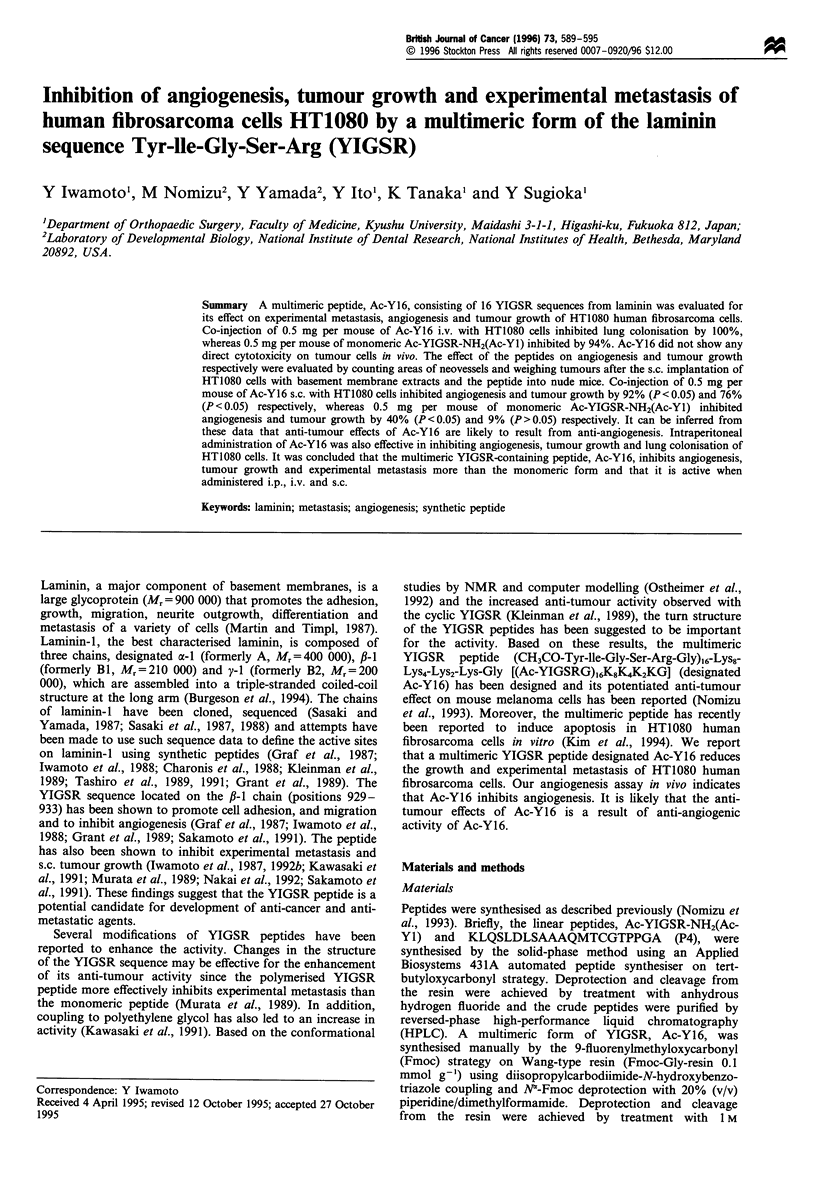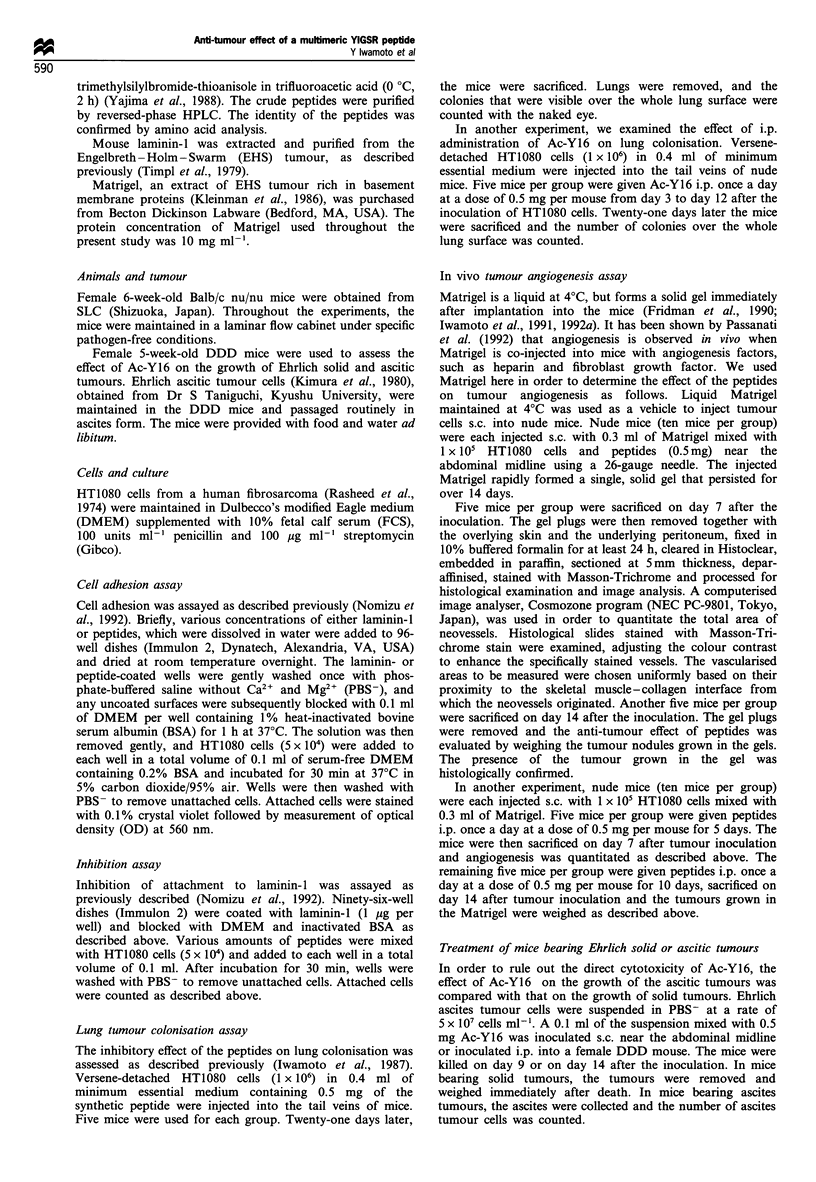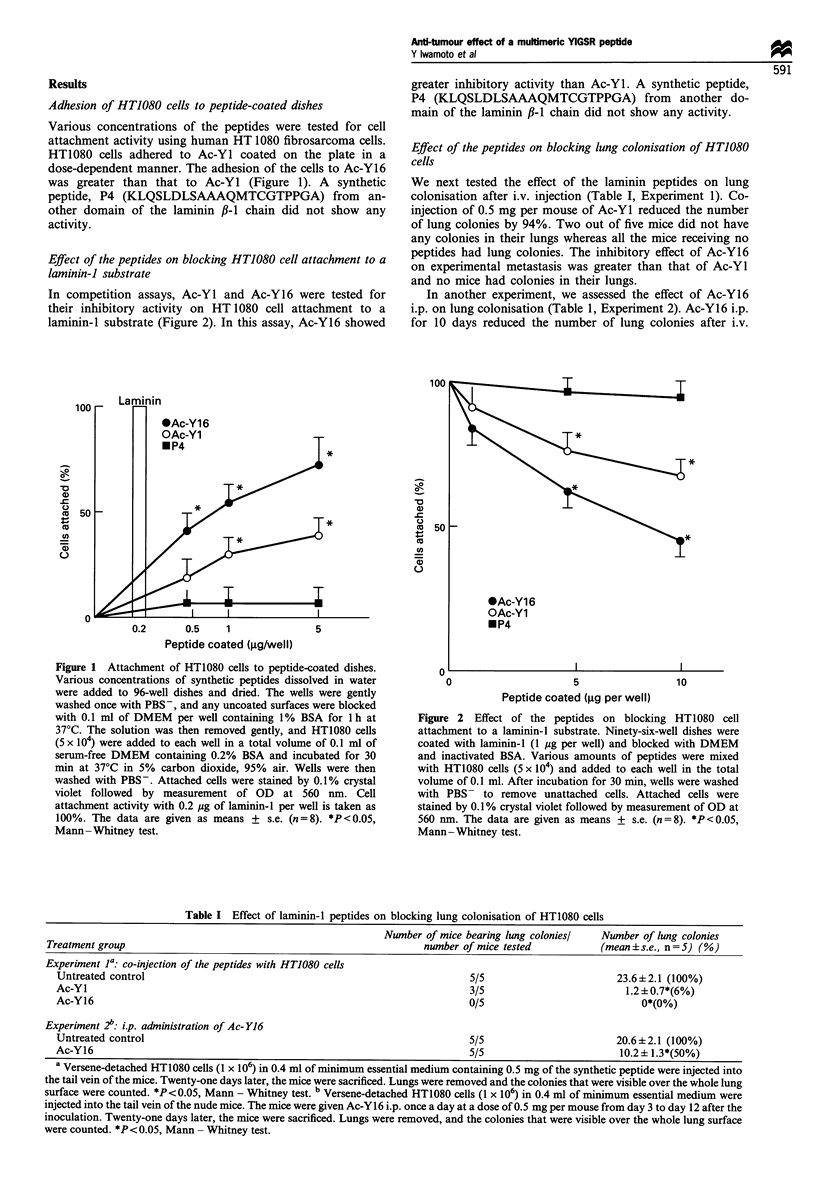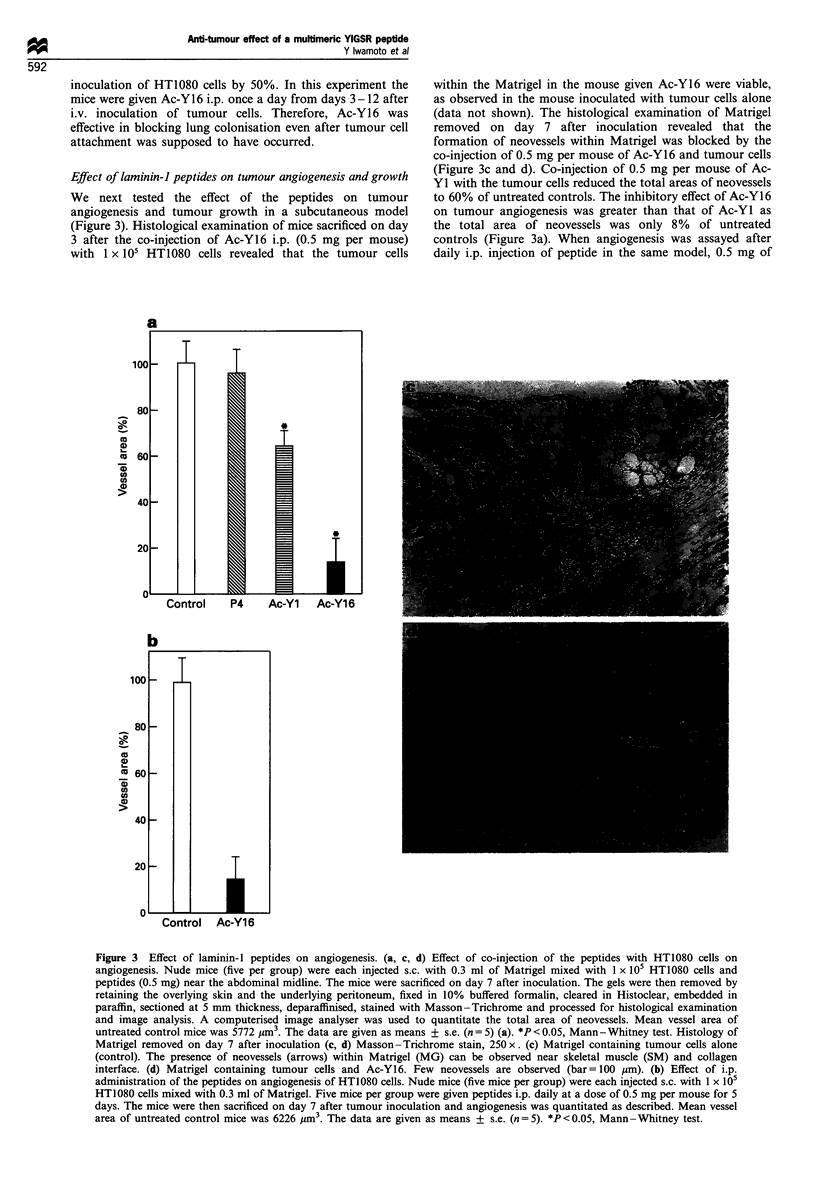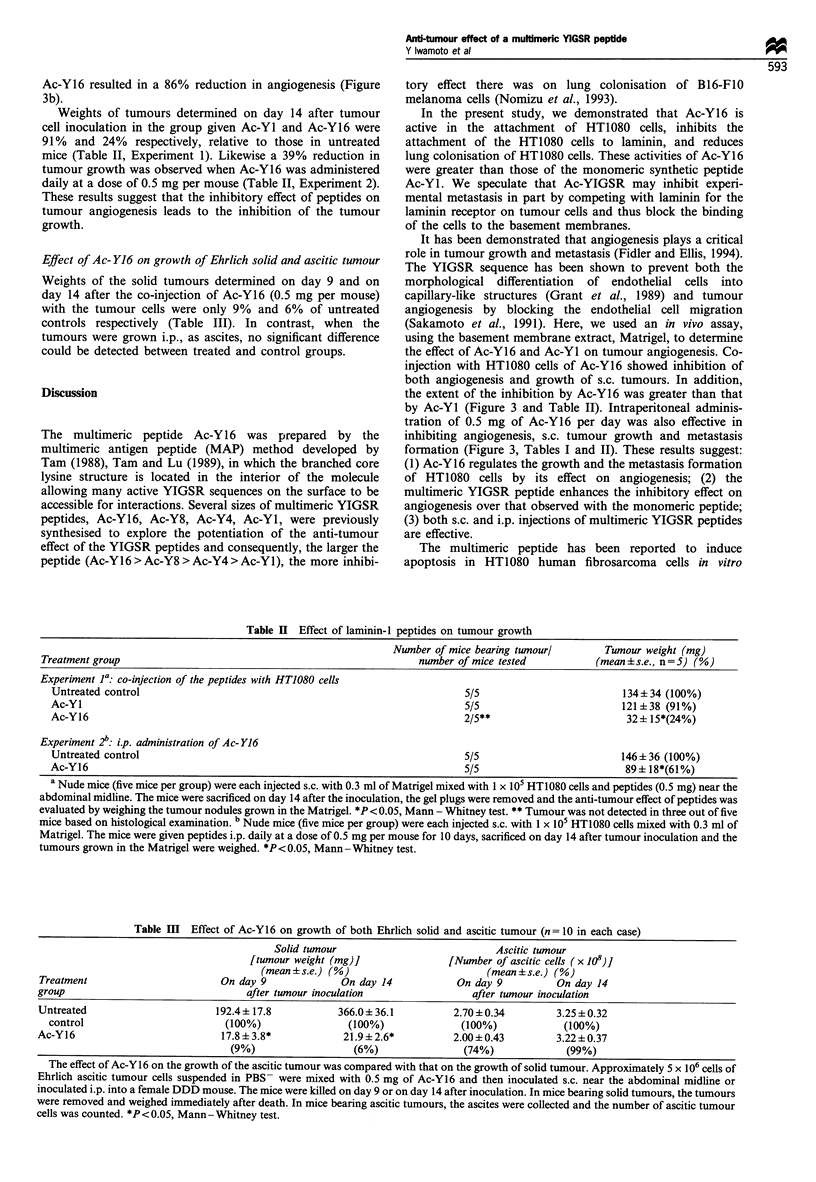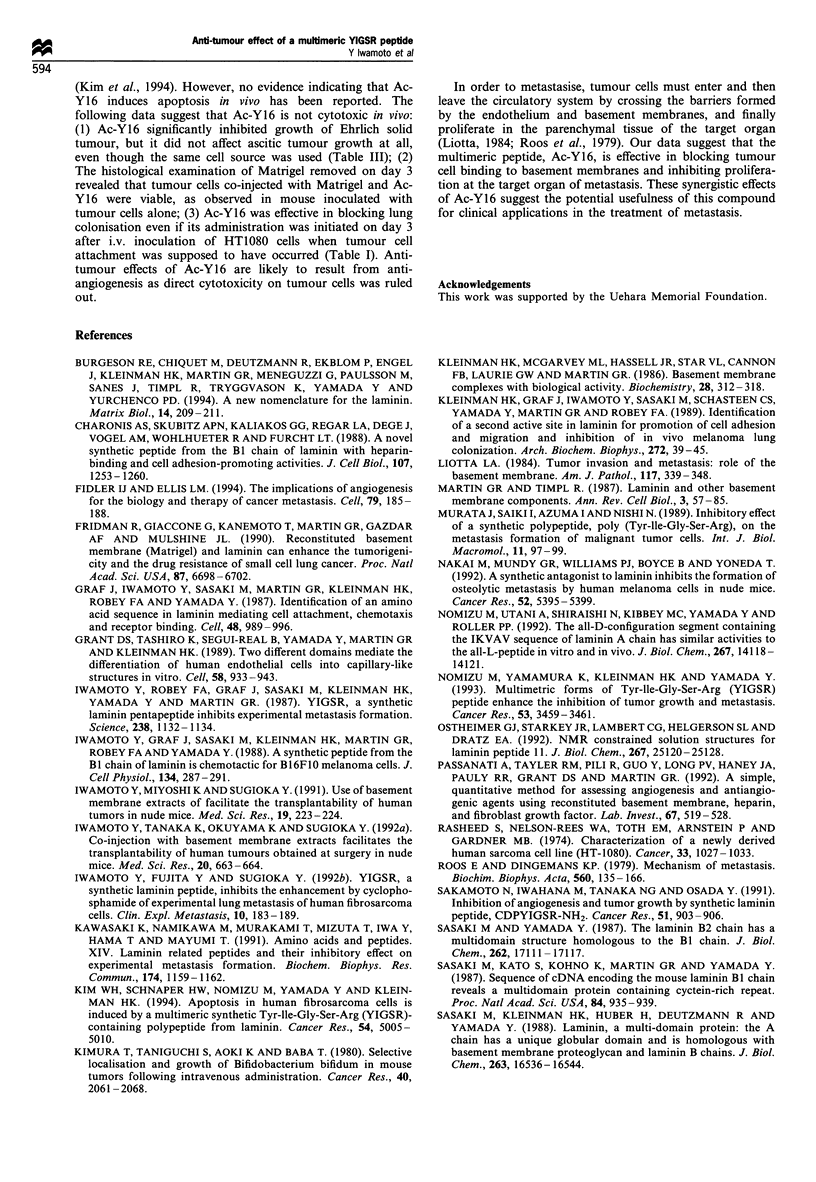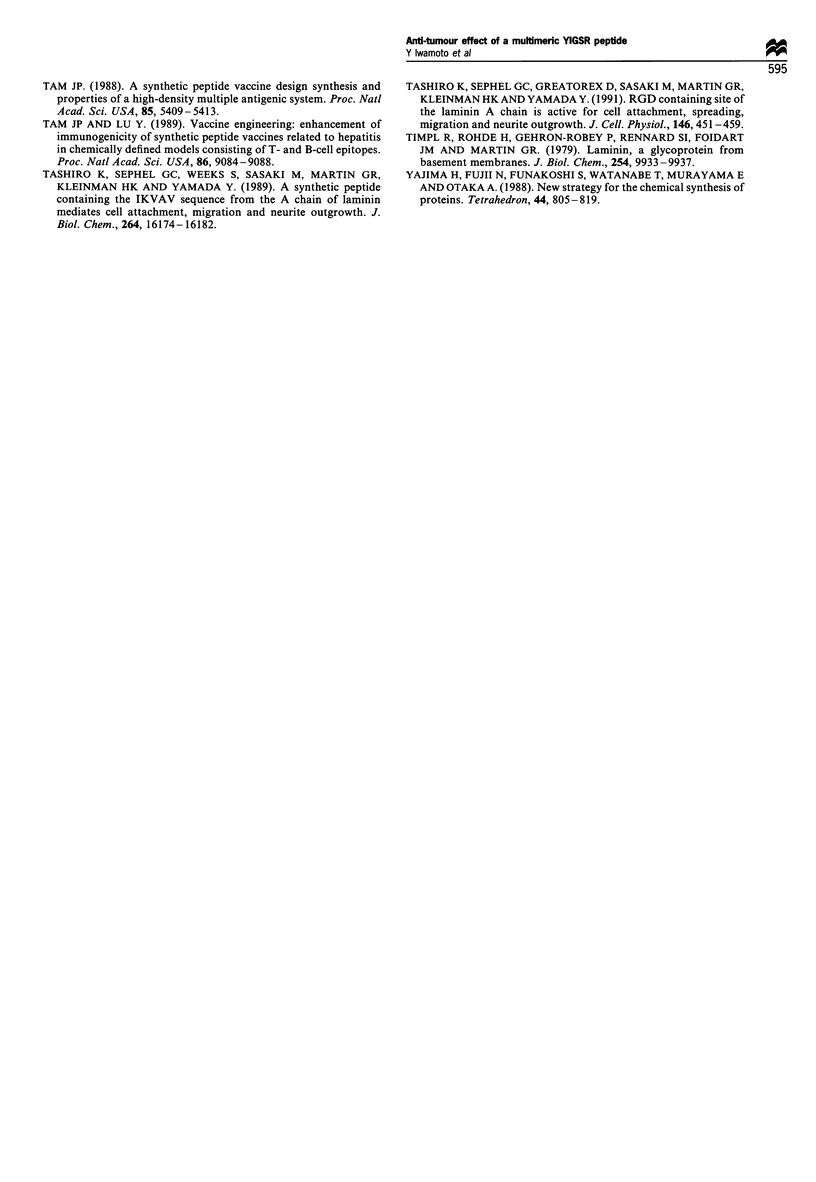# Inhibition of angiogenesis, tumour growth and experimental metastasis of human fibrosarcoma cells HT1080 by a multimeric form of the laminin sequence Tyr-Ile-Gly-Ser-Arg (YIGSR).

**DOI:** 10.1038/bjc.1996.102

**Published:** 1996-03

**Authors:** Y. Iwamoto, M. Nomizu, Y. Yamada, Y. Ito, K. Tanaka, Y. Sugioka

**Affiliations:** Department of Orthopaedic Surgery, Faculty of Medicine, Kyushu University, Fukuoka, Japan.

## Abstract

**Images:**


British Journal of Cancer (1996) 73, 589-595
© 1996 Stockton Press All rights reserved 0007-0920/96 $12.00

### Inhibition of angiogenesis, tumour growth and experimental metastasis of human fibrosarcoma cells HT1080 by a multimeric form of the laminin sequence Tyr-Ile-Gly-Ser-Arg (YIGSR)

Y Iwamoto[1], M Nomizu[2], Y Yamada[2], Y Ito[1], K Tanaka[1] and Y Sugioka[1]

[1]*Department of Orthopaedic Surgery, Faculty of Medicine, Kyushu University, Maidashi 3-1-1, Higashi-ku, Fukuoka 812, Japan;* [2]*Laboratory of Developmental Biology, National Institute of Dental Research, National Institutes of Health, Bethesda, Maryland 20892, USA.*

**Summary** A multimeric peptide, Ac-Y16, consisting of 16 YIGSR sequences from laminin was evaluated for its effect on experimental metastasis, angiogenesis and tumour growth of HT1080 human fibrosarcoma cells. Co-injection of 0.5 mg per mouse of Ac-Y16 i.v. with HT1080 cells inhibited lung colonisation by 100%, whereas 0.5 mg per mouse of monomeric Ac-YIGSR-$NH_2$(Ac-Y1) inhibited by 94%. Ac-Y16 did not show any direct cytotoxicity on tumour cells *in vivo*. The effect of the peptides on angiogenesis and tumour growth respectively were evaluated by counting areas of neovessels and weighing tumours after the s.c. implantation of HT1080 cells with basement membrane extracts and the peptide into nude mice. Co-injection of 0.5 mg per mouse of Ac-Y16 s.c. with HT1080 cells inhibited angiogenesis and tumour growth by 92% ($P<0.05$) and 76% ($P<0.05$) respectively, whereas 0.5 mg per mouse of monomeric Ac-YIGSR-$NH_2$(Ac-Y1) inhibited angiogenesis and tumour growth by 40% ($P<0.05$) and 9% ($P>0.05$) respectively. It can be inferred from these data that anti-tumour effects of Ac-Y16 are likely to result from anti-angiogenesis. Intraperitoneal administration of Ac-Y16 was also effective in inhibiting angiogenesis, tumour growth and lung colonisation of HT1080 cells. It was concluded that the multimeric YIGSR-containing peptide, Ac-Y16, inhibits angiogenesis, tumour growth and experimental metastasis more than the monomeric form and that it is active when administered i.p., i.v. and s.c.

**Keywords:** laminin; metastasis; angiogenesis; synthetic peptide

Laminin, a major component of basement membranes, is a large glycoprotein ($M_r=900\ 000$) that promotes the adhesion, growth, migration, neurite outgrowth, differentiation and metastasis of a variety of cells (Martin and Timpl, 1987). Laminin-1, the best characterised laminin, is composed of three chains, designated α-1 (formerly A, $M_r=400\ 000$), β-1 (formerly B1, $M_r=210\ 000$) and γ-1 (formerly B2, $M_r=200\ 000$), which are assembled into a triple-stranded coiled-coil structure at the long arm (Burgeson *et al.*, 1994). The chains of laminin-1 have been cloned, sequenced (Sasaki and Yamada, 1987; Sasaki *et al.*, 1987, 1988) and attempts have been made to use such sequence data to define the active sites on laminin-1 using synthetic peptides (Graf *et al.*, 1987; Iwamoto *et al.*, 1988; Charonis *et al.*, 1988; Kleinman *et al.*, 1989; Tashiro *et al.*, 1989, 1991; Grant *et al.*, 1989). The YIGSR sequence located on the β-1 chain (positions 929-933) has been shown to promote cell adhesion, and migration and to inhibit angiogenesis (Graf *et al.*, 1987; Iwamoto *et al.*, 1988; Grant *et al.*, 1989; Sakamoto *et al.*, 1991). The peptide has also been shown to inhibit experimental metastasis and s.c. tumour growth (Iwamoto *et al.*, 1987, 1992*b*; Kawasaki *et al.*, 1991; Murata *et al.*, 1989; Nakai *et al.*, 1992; Sakamoto *et al.*, 1991). These findings suggest that the YIGSR peptide is a potential candidate for development of anti-cancer and anti-metastatic agents.

Several modifications of YIGSR peptides have been reported to enhance the activity. Changes in the structure of the YIGSR sequence may be effective for the enhancement of its anti-tumour activity since the polymerised YIGSR peptide more effectively inhibits experimental metastasis than the monomeric peptide (Murata *et al.*, 1989). In addition, coupling to polyethylene glycol has also led to an increase in activity (Kawasaki *et al.*, 1991). Based on the conformational studies by NMR and computer modelling (Ostheimer *et al.*, 1992) and the increased anti-tumour activity observed with the cyclic YIGSR (Kleinman *et al.*, 1989), the turn structure of the YIGSR peptides has been suggested to be important for the activity. Based on these results, the multimeric YIGSR peptide ($CH_3CO$-Tyr-Ile-Gly-Ser-Arg-Gly)$_{16}$-Lys$_8$-Lys$_4$-Lys$_2$-Lys-Gly [(Ac-YIGSRG)$_{16}$K$_8$K$_4$K$_2$KG] (designated Ac-Y16) has been designed and its potentiated anti-tumour effect on mouse melanoma cells has been reported (Nomizu *et al.*, 1993). Moreover, the multimeric peptide has recently been reported to induce apoptosis in HT1080 human fibrosarcoma cells *in vitro* (Kim *et al.*, 1994). We report that a multimeric YIGSR peptide designated Ac-Y16 reduces the growth and experimental metastasis of HT1080 human fibrosarcoma cells. Our angiogenesis assay *in vivo* indicates that Ac-Y16 inhibits angiogenesis. It is likely that the anti-tumour effects of Ac-Y16 is a result of anti-angiogenic activity of Ac-Y16.

### Materials and methods

### *Materials*

Peptides were synthesised as described previously (Nomizu *et al.*, 1993). Briefly, the linear peptides, Ac-YIGSR-$NH_2$(Ac-Y1) and KLQSLDLSAAAQMTCGTPPGA (P4), were synthesised by the solid-phase method using an Applied Biosystems 431A automated peptide synthesiser on tert-butyloxycarbonyl strategy. Deprotection and cleavage from the resin were achieved by treatment with anhydrous hydrogen fluoride and the crude peptides were purified by reversed-phase high-performance liquid chromatography (HPLC). A multimeric form of YIGSR, Ac-Y16, was synthesised manually by the 9-fluorenylmethyloxycarbonyl (Fmoc) strategy on Wang-type resin (Fmoc-Gly-resin 0.1 mmol $g^{-1}$) using diisopropylcarbodiimide-*N*-hydroxybenzotriazole coupling and $N^{\alpha}$-Fmoc deprotection with 20% (v/v) piperidine/dimethylformamide. Deprotection and cleavage from the resin were achieved by treatment with 1 M

Correspondence: Y Iwamoto
Received 4 April 1995; revised 12 October 1995; accepted 27 October 1995

trimethylsilylbromide-thioanisole in trifluoroacetic acid (0 °C, 2 h) (Yajima *et al.*, 1988). The crude peptides were purified by reversed-phase HPLC. The identity of the peptides was confirmed by amino acid analysis.

Mouse laminin-1 was extracted and purified from the Engelbreth–Holm–Swarm (EHS) tumour, as described previously (Timpl *et al.*, 1979).

Matrigel, an extract of EHS tumour rich in basement membrane proteins (Kleinman *et al.*, 1986), was purchased from Becton Dickinson Labware (Bedford, MA, USA). The protein concentration of Matrigel used throughout the present study was 10 mg $ml^{-1}$.

*Animals and tumour*

Female 6-week-old Balb/c nu/nu mice were obtained from SLC (Shizuoka, Japan). Throughout the experiments, the mice were maintained in a laminar flow cabinet under specific pathogen-free conditions.

Female 5-week-old DDD mice were used to assess the effect of Ac-Y16 on the growth of Ehrlich solid and ascitic tumours. Ehrlich ascitic tumour cells (Kimura *et al.*, 1980), obtained from Dr S Taniguchi, Kyushu University, were maintained in the DDD mice and passaged routinely in ascites form. The mice were provided with food and water *ad libitum.*

*Cells and culture*

HT1080 cells from a human fibrosarcoma (Rasheed *et al.*, 1974) were maintained in Dulbecco's modified Eagle medium (DMEM) supplemented with 10% fetal calf serum (FCS), 100 units $ml^{-1}$ penicillin and 100 $\mu g$ $ml^{-1}$ streptomycin (Gibco).

*Cell adhesion assay*

Cell adhesion was assayed as described previously (Nomizu *et al.*, 1992). Briefly, various concentrations of either laminin-1 or peptides, which were dissolved in water were added to 96-well dishes (Immulon 2, Dynatech, Alexandria, VA, USA) and dried at room temperature overnight. The laminin- or peptide-coated wells were gently washed once with phosphate-buffered saline without $Ca^{2+}$ and $Mg^{2+}$ ($PBS^-$), and any uncoated surfaces were subsequently blocked with 0.1 ml of DMEM per well containing 1% heat-inactivated bovine serum albumin (BSA) for 1 h at 37°C. The solution was then removed gently, and HT1080 cells ($5 \times 10^4$) were added to each well in a total volume of 0.1 ml of serum-free DMEM containing 0.2% BSA and incubated for 30 min at 37°C in 5% carbon dioxide/95% air. Wells were then washed with $PBS^-$ to remove unattached cells. Attached cells were stained with 0.1% crystal violet followed by measurement of optical density (OD) at 560 nm.

*Inhibition assay*

Inhibition of attachment to laminin-1 was assayed as previously described (Nomizu *et al.*, 1992). Ninety-six-well dishes (Immulon 2) were coated with laminin-1 (1 $\mu g$ per well) and blocked with DMEM and inactivated BSA as described above. Various amounts of peptides were mixed with HT1080 cells ($5 \times 10^4$) and added to each well in a total volume of 0.1 ml. After incubation for 30 min, wells were washed with $PBS^-$ to remove unattached cells. Attached cells were counted as described above.

*Lung tumour colonisation assay*

The inhibitory effect of the peptides on lung colonisation was assessed as described previously (Iwamoto *et al.*, 1987). Versene-detached HT1080 cells ($1 \times 10^6$) in 0.4 ml of minimum essential medium containing 0.5 mg of the synthetic peptide were injected into the tail veins of mice. Five mice were used for each group. Twenty-one days later, the mice were sacrificed. Lungs were removed, and the colonies that were visible over the whole lung surface were counted with the naked eye.

In another experiment, we examined the effect of i.p. administration of Ac-Y16 on lung colonisation. Versene-detached HT1080 cells ($1 \times 10^6$) in 0.4 ml of minimum essential medium were injected into the tail veins of nude mice. Five mice per group were given Ac-Y16 i.p. once a day at a dose of 0.5 mg per mouse from day 3 to day 12 after the inoculation of HT1080 cells. Twenty-one days later the mice were sacrificed and the number of colonies over the whole lung surface was counted.

In vivo *tumour angiogenesis assay*

Matrigel is a liquid at 4°C, but forms a solid gel immediately after implantation into the mice (Fridman *et al.*, 1990; Iwamoto *et al.*, 1991, 1992*a*). It has been shown by Passanati *et al.* (1992) that angiogenesis is observed *in vivo* when Matrigel is co-injected into mice with angiogenesis factors, such as heparin and fibroblast growth factor. We used Matrigel here in order to determine the effect of the peptides on tumour angiogenesis as follows. Liquid Matrigel maintained at 4°C was used as a vehicle to inject tumour cells s.c. into nude mice. Nude mice (ten mice per group) were each injected s.c. with 0.3 ml of Matrigel mixed with $1 \times 10^5$ HT1080 cells and peptides (0.5 mg) near the abdominal midline using a 26-gauge needle. The injected Matrigel rapidly formed a single, solid gel that persisted for over 14 days.

Five mice per group were sacrificed on day 7 after the inoculation. The gel plugs were then removed together with the overlying skin and the underlying peritoneum, fixed in 10% buffered formalin for at least 24 h, cleared in Histoclear, embedded in paraffin, sectioned at 5 mm thickness, deparaffinised, stained with Masson-Trichrome and processed for histological examination and image analysis. A computerised image analyser, Cosmozone program (NEC PC-9801, Tokyo, Japan), was used in order to quantitate the total area of neovessels. Histological slides stained with Masson-Trichrome stain were examined, adjusting the colour contrast to enhance the specifically stained vessels. The vascularised areas to be measured were chosen uniformly based on their proximity to the skeletal muscle–collagen interface from which the neovessels originated. Another five mice per group were sacrificed on day 14 after the inoculation. The gel plugs were removed and the anti-tumour effect of peptides was evaluated by weighing the tumour nodules grown in the gels. The presence of the tumour grown in the gel was histologically confirmed.

In another experiment, nude mice (ten mice per group) were each injected s.c. with $1 \times 10^5$ HT1080 cells mixed with 0.3 ml of Matrigel. Five mice per group were given peptides i.p. once a day at a dose of 0.5 mg per mouse for 5 days. The mice were then sacrificed on day 7 after tumour inoculation and angiogenesis was quantitated as described above. The remaining five mice per group were given peptides i.p. once a day at a dose of 0.5 mg per mouse for 10 days, sacrificed on day 14 after tumour inoculation and the tumours grown in the Matrigel were weighed as described above.

*Treatment of mice bearing Ehrlich solid or ascitic tumours*

In order to rule out the direct cytotoxicity of Ac-Y16, the effect of Ac-Y16 on the growth of the ascitic tumours was compared with that on the growth of solid tumours. Ehrlich ascites tumour cells were suspended in $PBS^-$ at a rate of $5 \times 10^7$ cells $ml^{-1}$. A 0.1 ml of the suspension mixed with 0.5 mg Ac-Y16 was inoculated s.c. near the abdominal midline or inoculated i.p. into a female DDD mouse. The mice were killed on day 9 or on day 14 after the inoculation. In mice bearing solid tumours, the tumours were removed and weighed immediately after death. In mice bearing ascites tumours, the ascites were collected and the number of ascites tumour cells was counted.

### Results

### *Adhesion of HT1080 cells to peptide-coated dishes*

Various concentrations of the peptides were tested for cell attachment activity using human HT 1080 fibrosarcoma cells. HT1080 cells adhered to Ac-Y1 coated on the plate in a dose-dependent manner. The adhesion of the cells to Ac-Y16 was greater than that to Ac-Y1 (Figure 1). A synthetic peptide, P4 (KLQSLDLSAAAQMTCGTPPGA) from another domain of the laminin $\beta$-1 chain did not show any activity.

### *Effect of the peptides on blocking HT1080 cell attachment to a laminin-1 substrate*

In competition assays, Ac-Y1 and Ac-Y16 were tested for their inhibitory activity on HT 1080 cell attachment to a laminin-1 substrate (Figure 2). In this assay, Ac-Y16 showed greater inhibitory activity than Ac-Y1. A synthetic peptide, P4 (KLQSLDLSAAAQMTCGTPPGA) from another domain of the laminin $\beta$-1 chain did not show any activity.

### *Effect of the peptides on blocking lung colonisation of HT1080 cells*

We next tested the effect of the laminin peptides on lung colonisation after i.v. injection (Table I, Experiment 1). Co-injection of 0.5 mg per mouse of Ac-Y1 reduced the number of lung colonies by 94%. Two out of five mice did not have any colonies in their lungs whereas all the mice receiving no peptides had lung colonies. The inhibitory effect of Ac-Y16 on experimental metastasis was greater than that of Ac-Y1 and no mice had colonies in their lungs.

In another experiment, we assessed the effect of Ac-Y16 i.p. on lung colonisation (Table 1, Experiment 2). Ac-Y16 i.p. for 10 days reduced the number of lung colonies after i.v.

**Figure 1** Attachment of HT1080 cells to peptide-coated dishes. Various concentrations of synthetic peptides dissolved in water were added to 96-well dishes and dried. The wells were gently washed once with PBS$^-$, and any uncoated surfaces were blocked with 0.1 ml of DMEM per well containing 1% BSA for 1 h at 37°C. The solution was then removed gently, and HT1080 cells ($5 \times 10^4$) were added to each well in a total volume of 0.1 ml of serum-free DMEM containing 0.2% BSA and incubated for 30 min at 37°C in 5% carbon dioxide, 95% air. Wells were then washed with PBS$^-$. Attached cells were stained by 0.1% crystal violet followed by measurement of OD at 560 nm. Cell attachment activity with 0.2 $\mu$g of laminin-1 per well is taken as 100%. The data are given as means $\pm$ s.e. ($n=8$). *$P<0.05$, Mann–Whitney test.

**Figure 2** Effect of the peptides on blocking HT1080 cell attachment to a laminin-1 substrate. Ninety-six-well dishes were coated with laminin-1 (1 $\mu$g per well) and blocked with DMEM and inactivated BSA. Various amounts of peptides were mixed with HT1080 cells ($5 \times 10^4$) and added to each well in the total volume of 0.1 ml. After incubation for 30 min, wells were washed with PBS$^-$ to remove unattached cells. Attached cells were stained by 0.1% crystal violet followed by measurement of OD at 560 nm. The data are given as means $\pm$ s.e. ($n=8$). *$P<0.05$, Mann–Whitney test.

**Table I** Effect of laminin-1 peptides on blocking lung colonisation of HT1080 cells

| *Treatment group* | *Number of mice bearing lung colonies/ number of mice tested* | *Number of lung colonies (mean ± s.e., n = 5) (%)* |
|---|---|---|
| *Experiment 1[a]: co-injection of the peptides with HT1080 cells* | | |
| Untreated control | 5/5 | 23.6 ± 2.1 (100%) |
| Ac-Y1 | 3/5 | 1.2 ± 0.7*(6%) |
| Ac-Y16 | 0/5 | 0*(0%) |
| *Experiment 2[b]: i.p. administration of Ac-Y16* | | |
| Untreated control | 5/5 | 20.6 ± 2.1 (100%) |
| Ac-Y16 | 5/5 | 10.2 ± 1.3*(50%) |

[a] Versene-detached HT1080 cells ($1 \times 10^6$) in 0.4 ml of minimum essential medium containing 0.5 mg of the synthetic peptide were injected into the tail vein of the mice. Twenty-one days later, the mice were sacrificed. Lungs were removed and the colonies that were visible over the whole lung surface were counted. *$P<0.05$, Mann – Whitney test. [b] Versene-detached HT1080 cells ($1 \times 10^6$) in 0.4 ml of minimum essential medium were injected into the tail vein of the nude mice. The mice were given Ac-Y16 i.p. once a day at a dose of 0.5 mg per mouse from day 3 to day 12 after the inoculation. Twenty-one days later, the mice were sacrificed. Lungs were removed, and the colonies that were visible over the whole lung surface were counted. *$P<0.05$, Mann – Whitney test.

inoculation of HT1080 cells by 50%. In this experiment the mice were given Ac-Y16 i.p. once a day from days 3–12 after i.v. inoculation of tumour cells. Therefore, Ac-Y16 was effective in blocking lung colonisation even after tumour cell attachment was supposed to have occurred.

*Effect of laminin-1 peptides on tumour angiogenesis and growth*

We next tested the effect of the peptides on tumour angiogenesis and tumour growth in a subcutaneous model (Figure 3). Histological examination of mice sacrificed on day 3 after the co-injection of Ac-Y16 i.p. (0.5 mg per mouse) with $1 \times 10^5$ HT1080 cells revealed that the tumour cells within the Matrigel in the mouse given Ac-Y16 were viable, as observed in the mouse inoculated with tumour cells alone (data not shown). The histological examination of Matrigel removed on day 7 after inoculation revealed that the formation of neovessels within Matrigel was blocked by the co-injection of 0.5 mg per mouse of Ac-Y16 and tumour cells (Figure 3c and d). Co-injection of 0.5 mg per mouse of Ac-Y1 with the tumour cells reduced the total areas of neovessels to 60% of untreated controls. The inhibitory effect of Ac-Y16 on tumour angiogenesis was greater than that of Ac-Y1 as the total area of neovessels was only 8% of untreated controls (Figure 3a). When angiogenesis was assayed after daily i.p. injection of peptide in the same model, 0.5 mg of

**Figure 3** Effect of laminin-1 peptides on angiogenesis. (**a**, **c**, **d**) Effect of co-injection of the peptides with HT1080 cells on angiogenesis. Nude mice (five per group) were each injected s.c. with 0.3 ml of Matrigel mixed with $1 \times 10^5$ HT1080 cells and peptides (0.5 mg) near the abdominal midline. The mice were sacrificed on day 7 after inoculation. The gels were then removed by retaining the overlying skin and the underlying peritoneum, fixed in 10% buffered formalin, cleared in Histoclear, embedded in paraffin, sectioned at 5 mm thickness, deparaffinised, stained with Masson–Trichrome and processed for histological examination and image analysis. A computerised image analyser was used to quantitate the total area of neovessels. Mean vessel area of untreated control mice was 5772 $\mu m^3$. The data are given as means $\pm$ s.e. ($n=5$) (**a**). *$P<0.05$, Mann–Whitney test. Histology of Matrigel removed on day 7 after inoculation (**c**, **d**) Masson–Trichrome stain, 250×. (**c**) Matrigel containing tumour cells alone (control). The presence of neovessels (arrows) within Matrigel (MG) can be observed near skeletal muscle (SM) and collagen interface. (**d**) Matrigel containing tumour cells and Ac-Y16. Few neovessels are observed (bar = 100 $\mu m$). (**b**) Effect of i.p. administration of the peptides on angiogenesis of HT1080 cells. Nude mice (five mice per group) were each injected s.c. with $1 \times 10^5$ HT1080 cells mixed with 0.3 ml of Matrigel. Five mice per group were given peptides i.p. daily at a dose of 0.5 mg per mouse for 5 days. The mice were then sacrificed on day 7 after tumour inoculation and angiogenesis was quantitated as described. Mean vessel area of untreated control mice was 6226 $\mu m^3$. The data are given as means $\pm$ s.e. ($n=5$). *$P<0.05$, Mann–Whitney test.

Ac-Y16 resulted in a 86% reduction in angiogenesis (Figure 3b).

Weights of tumours determined on day 14 after tumour cell inoculation in the group given Ac-Y1 and Ac-Y16 were 91% and 24% respectively, relative to those in untreated mice (Table II, Experiment 1). Likewise a 39% reduction in tumour growth was observed when Ac-Y16 was administered daily at a dose of 0.5 mg per mouse (Table II, Experiment 2). These results suggest that the inhibitory effect of peptides on tumour angiogenesis leads to the inhibition of the tumour growth.

*Effect of Ac-Y16 on growth of Ehrlich solid and ascitic tumour*

Weights of the solid tumours determined on day 9 and on day 14 after the co-injection of Ac-Y16 (0.5 mg per mouse) with the tumour cells were only 9% and 6% of untreated controls respectively (Table III). In contrast, when the tumours were grown i.p., as ascites, no significant difference could be detected between treated and control groups.

### Discussion

The multimeric peptide Ac-Y16 was prepared by the multimeric antigen peptide (MAP) method developed by Tam (1988), Tam and Lu (1989), in which the branched core lysine structure is located in the interior of the molecule allowing many active YIGSR sequences on the surface to be accessible for interactions. Several sizes of multimeric YIGSR peptides, Ac-Y16, Ac-Y8, Ac-Y4, Ac-Y1, were previously synthesised to explore the potentiation of the anti-tumour effect of the YIGSR peptides and consequently, the larger the peptide (Ac-Y16 > Ac-Y8 > Ac-Y4 > Ac-Y1), the more inhibitory effect there was on lung colonisation of B16-F10 melanoma cells (Nomizu *et al.*, 1993).

In the present study, we demonstrated that Ac-Y16 is active in the attachment of HT1080 cells, inhibits the attachment of the HT1080 cells to laminin, and reduces lung colonisation of HT1080 cells. These activities of Ac-Y16 were greater than those of the monomeric synthetic peptide Ac-Y1. We speculate that Ac-YIGSR may inhibit experimental metastasis in part by competing with laminin for the laminin receptor on tumour cells and thus block the binding of the cells to the basement membranes.

It has been demonstrated that angiogenesis plays a critical role in tumour growth and metastasis (Fidler and Ellis, 1994). The YIGSR sequence has been shown to prevent both the morphological differentiation of endothelial cells into capillary-like structures (Grant *et al.*, 1989) and tumour angiogenesis by blocking the endothelial cell migration (Sakamoto *et al.*, 1991). Here, we used an *in vivo* assay, using the basement membrane extract, Matrigel, to determine the effect of Ac-Y16 and Ac-Y1 on tumour angiogenesis. Co-injection with HT1080 cells of Ac-Y16 showed inhibition of both angiogenesis and growth of s.c. tumours. In addition, the extent of the inhibition by Ac-Y16 was greater than that by Ac-Y1 (Figure 3 and Table II). Intraperitoneal administration of 0.5 mg of Ac-Y16 per day was also effective in inhibiting angiogenesis, s.c. tumour growth and metastasis formation (Figure 3, Tables I and II). These results suggest: (1) Ac-Y16 regulates the growth and the metastasis formation of HT1080 cells by its effect on angiogenesis; (2) the multimeric YIGSR peptide enhances the inhibitory effect on angiogenesis over that observed with the monomeric peptide; (3) both s.c. and i.p. injections of multimeric YIGSR peptides are effective.

The multimeric peptide has been reported to induce apoptosis in HT1080 human fibrosarcoma cells *in vitro*

**Table II** Effect of laminin-1 peptides on tumour growth

| Treatment group | *Number of mice bearing tumour/ number of mice tested* | *Tumour weight (mg) (mean ± s.e., n = 5) (%)* |
|---|---|---|
| *Experiment 1[a]: co-injection of the peptides with HT1080 cells* | | |
| Untreated control | 5/5 | 134 ± 34 (100%) |
| Ac-Y1 | 5/5 | 121 ± 38 (91%) |
| Ac-Y16 | 2/5** | 32 ± 15*(24%) |
| *Experiment 2[b]: i.p. administration of Ac-Y16* | | |
| Untreated control | 5/5 | 146 ± 36 (100%) |
| Ac-Y16 | 5/5 | 89 ± 18*(61%) |

[a] Nude mice (five mice per group) were each injected s.c. with 0.3 ml of Matrigel mixed with $1 \times 10^5$ HT1080 cells and peptides (0.5 mg) near the abdominal midline. The mice were sacrificed on day 14 after the inoculation, the gel plugs were removed and the anti-tumour effect of peptides was evaluated by weighing the tumour nodules grown in the Matrigel. *$P < 0.05$, Mann – Whitney test. ** Tumour was not detected in three out of five mice based on histological examination. [b] Nude mice (five mice per group) were each injected s.c. with $1 \times 10^5$ HT1080 cells mixed with 0.3 ml of Matrigel. The mice were given peptides i.p. daily at a dose of 0.5 mg per mouse for 10 days, sacrificed on day 14 after tumour inoculation and the tumours grown in the Matrigel were weighed. *$P < 0.05$, Mann – Whitney test.

**Table III** Effect of Ac-Y16 on growth of both Ehrlich solid and ascitic tumour ($n = 10$ in each case)

| Treatment group | *Solid tumour [tumour weight (mg)] (mean ± s.e.) (%)* | | *Ascitic tumour [Number of ascitic cells ($\times 10^8$)] (mean ± s.e.) (%)* | |
|---|---|---|---|---|
| | *On day 9 after tumour inoculation* | *On day 14 after tumour inoculation* | *On day 9 after tumour inoculation* | *On day 14 after tumour inoculation* |
| Untreated control | 192.4 ± 17.8 (100%) | 366.0 ± 36.1 (100%) | 2.70 ± 0.34 (100%) | 3.25 ± 0.32 (100%) |
| Ac-Y16 | 17.8 ± 3.8* (9%) | 21.9 ± 2.6* (6%) | 2.00 ± 0.43 (74%) | 3.22 ± 0.37 (99%) |

The effect of Ac-Y16 on the growth of the ascitic tumour was compared with that on the growth of solid tumour. Approximately $5 \times 10^6$ cells of Ehrlich ascitic tumour cells suspended in $PBS^-$ were mixed with 0.5 mg of Ac-Y16 and then inoculated s.c. near the abdominal midline or inoculated i.p. into a female DDD mouse. The mice were killed on day 9 or on day 14 after inoculation. In mice bearing solid tumours, the tumours were removed and weighed immediately after death. In mice bearing ascitic tumours, the ascites were collected and the number of ascitic tumour cells was counted. *$P < 0.05$, Mann – Whitney test.

(Kim *et al.*, 1994). However, no evidence indicating that Ac-Y16 induces apoptosis *in vivo* has been reported. The following data suggest that Ac-Y16 is not cytotoxic *in vivo*: (1) Ac-Y16 significantly inhibited growth of Ehrlich solid tumour, but it did not affect ascitic tumour growth at all, even though the same cell source was used (Table III); (2) The histological examination of Matrigel removed on day 3 revealed that tumour cells co-injected with Matrigel and Ac-Y16 were viable, as observed in mouse inoculated with tumour cells alone; (3) Ac-Y16 was effective in blocking lung colonisation even if its administration was initiated on day 3 after i.v. inoculation of HT1080 cells when tumour cell attachment was supposed to have occurred (Table I). Anti-tumour effects of Ac-Y16 are likely to result from anti-angiogenesis as direct cytotoxicity on tumour cells was ruled out.

In order to metastasise, tumour cells must enter and then leave the circulatory system by crossing the barriers formed by the endothelium and basement membranes, and finally proliferate in the parenchymal tissue of the target organ (Liotta, 1984; Roos *et al.*, 1979). Our data suggest that the multimeric peptide, Ac-Y16, is effective in blocking tumour cell binding to basement membranes and inhibiting proliferation at the target organ of metastasis. These synergistic effects of Ac-Y16 suggest the potential usefulness of this compound for clinical applications in the treatment of metastasis.

**Acknowledgements**
This work was supported by the Uehara Memorial Foundation.

### References

BURGESON RE, CHIQUET M, DEUTZMANN R, EKBLOM P, ENGEL J, KLEINMAN HK, MARTIN GR, MENEGUZZI G, PAULSSON M, SANES J, TIMPL R, TRYGGVASON K, YAMADA Y AND YURCHENCO PD. (1994). A new nomenclature for the laminin. *Matrix Biol.*, **14**, 209–211.

CHARONIS AS, SKUBITZ APN, KALIAKOS GG, REGAR LA, DEGE J, VOGEL AM, WOHLHUETER R AND FURCHT LT. (1988). A novel synthetic peptide from the B1 chain of laminin with heparin-binding and cell adhesion-promoting activities. *J. Cell Biol.*, **107**, 1253–1260.

FIDLER IJ AND ELLIS LM. (1994). The implications of angiogenesis for the biology and therapy of cancer metastasis. *Cell*, **79**, 185–188.

FRIDMAN R, GIACCONE G, KANEMOTO T, MARTIN GR, GAZDAR AF AND MULSHINE JL. (1990). Reconstituted basement membrane (Matrigel) and laminin can enhance the tumorigenicity and the drug resistance of small cell lung cancer. *Proc. Natl Acad. Sci. USA*, **87**, 6698–6702.

GRAF J, IWAMOTO Y, SASAKI M, MARTIN GR, KLEINMAN HK, ROBEY FA AND YAMADA Y. (1987). Identification of an amino acid sequence in laminin mediating cell attachment, chemotaxis and receptor binding. *Cell*, **48**, 989–996.

GRANT DS, TASHIRO K, SEGUI-REAL B, YAMADA Y, MARTIN GR AND KLEINMAN HK. (1989). Two different domains mediate the differentiation of human endothelial cells into capillary-like structures in vitro. *Cell*, **58**, 933–943.

IWAMOTO Y, ROBEY FA, GRAF J, SASAKI M, KLEINMAN HK, YAMADA Y AND MARTIN GR. (1987). YIGSR, a synthetic laminin pentapeptide inhibits experimental metastasis formation. *Science*, **238**, 1132–1134.

IWAMOTO Y, GRAF J, SASAKI M, KLEINMAN HK, MARTIN GR, ROBEY FA AND YAMADA Y. (1988). A synthetic peptide from the B1 chain of laminin is chemotactic for B16F10 melanoma cells. *J. Cell Physiol.*, **134**, 287–291.

IWAMOTO Y, MIYOSHI K AND SUGIOKA Y. (1991). Use of basement membrane extracts of facilitate the transplantability of human tumors in nude mice. *Med. Sci. Res.*, **19**, 223–224.

IWAMOTO Y, TANAKA K, OKUYAMA K AND SUGIOKA Y. (1992*a*). Co-injection with basement membrane extracts facilitates the transplantability of human tumours obtained at surgery in nude mice. *Med. Sci. Res.*, **20**, 663–664.

IWAMOTO Y, FUJITA Y AND SUGIOKA Y. (1992*b*). YIGSR, a synthetic laminin peptide, inhibits the enhancement by cyclophosphamide of experimental lung metastasis of human fibrosarcoma cells. *Clin. Expl. Metastasis*, **10**, 183–189.

KAWASAKI K, NAMIKAWA M, MURAKAMI T, MIZUTA T, IWA Y, HAMA T AND MAYUMI T. (1991). Amino acids and peptides. XIV. Laminin related peptides and their inhibitory effect on experimental metastasis formation. *Biochem. Biophys. Res. Commun.*, **174**, 1159–1162.

KIM WH, SCHNAPER HW, NOMIZU M, YAMADA Y AND KLEINMAN HK. (1994). Apoptosis in human fibrosarcoma cells is induced by a multimeric synthetic Tyr-Ile-Gly-Ser-Arg (YIGSR)-containing polypeptide from laminin. *Cancer Res.*, **54**, 5005–5010.

KIMURA T, TANIGUCHI S, AOKI K AND BABA T. (1980). Selective localisation and growth of Bifidobacterium bifidum in mouse tumors following intravenous administration. *Cancer Res.*, **40**, 2061–2068.

KLEINMAN HK, MCGARVEY ML, HASSELL JR, STAR VL, CANNON FB, LAURIE GW AND MARTIN GR. (1986). Basement membrane complexes with biological activity. *Biochemistry*, **28**, 312–318.

KLEINMAN HK, GRAF J, IWAMOTO Y, SASAKI M, SCHASTEEN CS, YAMADA Y, MARTIN GR AND ROBEY FA. (1989). Identification of a second active site in laminin for promotion of cell adhesion and migration and inhibition of in vivo melanoma lung colonization. *Arch. Biochem. Biophys.*, **272**, 39–45.

LIOTTA LA. (1984). Tumor invasion and metastasis: role of the basement membrane. *Am. J. Pathol.*, **117**, 339–348.

MARTIN GR AND TIMPL R. (1987). Laminin and other basement membrane components. *Ann. Rev. Cell Biol.*, **3**, 57–85.

MURATA J, SAIKI I, AZUMA I AND NISHI N. (1989). Inhibitory effect of a synthetic polypeptide, poly (Tyr-Ile-Gly-Ser-Arg), on the metastasis formation of malignant tumor cells. *Int. J. Biol. Macromol.*, **11**, 97–99.

NAKAI M, MUNDY GR, WILLIAMS PJ, BOYCE B AND YONEDA T. (1992). A synthetic antagonist to laminin inhibits the formation of osteolytic metastasis by human melanoma cells in nude mice. *Cancer Res.*, **52**, 5395–5399.

NOMIZU M, UTANI A, SHIRAISHI N, KIBBEY MC, YAMADA Y AND ROLLER PP. (1992). The all-D-configuration segment containing the IKVAV sequence of laminin A chain has similar activities to the all-L-peptide in vitro and in vivo. *J. Biol. Chem.*, **267**, 14118–14121.

NOMIZU M, YAMAMURA K, KLEINMAN HK AND YAMADA Y. (1993). Multimetric forms of Tyr-Ile-Gly-Ser-Arg (YIGSR) peptide enhance the inhibition of tumor growth and metastasis. *Cancer Res.*, **53**, 3459–3461.

OSTHEIMER GJ, STARKEY JR, LAMBERT CG, HELGERSON SL AND DRATZ EA. (1992). NMR constrained solution structures for laminin peptide 11. *J. Biol. Chem.*, **267**, 25120–25128.

PASSANATI A, TAYLER RM, PILI R, GUO Y, LONG PV, HANEY JA, PAULY RR, GRANT DS AND MARTIN GR. (1992). A simple, quantitative method for assessing angiogenesis and antiangiogenic agents using reconstituted basement membrane, heparin, and fibroblast growth factor. *Lab. Invest.*, **67**, 519–528.

RASHEED S, NELSON-REES WA, TOTH EM, ARNSTEIN P AND GARDNER MB. (1974). Characterization of a newly derived human sarcoma cell line (HT-1080). *Cancer*, **33**, 1027–1033.

ROOS E AND DINGEMANS KP. (1979). Mechanism of metastasis. *Biochim. Biophys. Acta*, **560**, 135–166.

SAKAMOTO N, IWAHANA M, TANAKA NG AND OSADA Y. (1991). Inhibition of angiogenesis and tumor growth by synthetic laminin peptide, CDPYIGSR-$NH_2$. *Cancer Res.*, **51**, 903–906.

SASAKI M AND YAMADA Y. (1987). The laminin B2 chain has a multidomain structure homologous to the B1 chain. *J. Biol. Chem.*, **262**, 17111–17117.

SASAKI M, KATO S, KOHNO K, MARTIN GR AND YAMADA Y. (1987). Sequence of cDNA encoding the mouse laminin B1 chain reveals a multidomain protein containing cyctein-rich repeat. *Proc. Natl Acad. Sci. USA*, **84**, 935–939.

SASAKI M, KLEINMAN HK, HUBER H, DEUTZMANN R AND YAMADA Y. (1988). Laminin, a multi-domain protein: the A chain has a unique globular domain and is homologous with basement membrane proteoglycan and laminin B chains. *J. Biol. Chem.*, **263**, 16536–16544.

TAM JP. (1988). A synthetic peptide vaccine design synthesis and properties of a high-density multiple antigenic system. *Proc. Natl Acad. Sci. USA*, **85**, 5409–5413.

TAM JP AND LU Y. (1989). Vaccine engineering: enhancement of immunogenicity of synthetic peptide vaccines related to hepatitis in chemically defined models consisting of T- and B-cell epitopes. *Proc. Natl Acad. Sci. USA*, **86**, 9084–9088.

TASHIRO K, SEPHEL GC, WEEKS S, SASAKI M, MARTIN GR, KLEINMAN HK AND YAMADA Y. (1989). A synthetic peptide containing the IKVAV sequence from the A chain of laminin mediates cell attachment, migration and neurite outgrowth. *J. Biol. Chem.*, **264**, 16174–16182.

TASHIRO K, SEPHEL GC, GREATOREX D, SASAKI M, MARTIN GR, KLEINMAN HK AND YAMADA Y. (1991). RGD containing site of the laminin A chain is active for cell attachment, spreading, migration and neurite outgrowth. *J. Cell. Physiol.*, **146**, 451–459.

TIMPL R, ROHDE H, GEHRON-ROBEY P, RENNARD SI, FOIDART JM AND MARTIN GR. (1979). Laminin, a glycoprotein from basement membranes. *J. Biol. Chem.*, **254**, 9933–9937.

YAJIMA H, FUJII N, FUNAKOSHI S, WATANABE T, MURAYAMA E AND OTAKA A. (1988). New strategy for the chemical synthesis of proteins. *Tetrahedron*, **44**, 805–819.